# Security as a Natural Law: A Quantum-Inspired Hypothesis for Information Persistence

**DOI:** 10.3390/e28070770

**Published:** 2026-07-07

**Authors:** Pete Herzog, Michael Sletten, Šarūnas Grigaliūnas, Rasa Brūzgienė

**Affiliations:** 1 The Institute for Security and Open Methodologies (ISECOM), Apartat de Correus 134, 08440 Cardedeu, Spain; 2 College of Business, Information Studies, and Technology, Dominican University, River Forest, IL 60305, USA; msletten@dom.edu; 3 Cyber Security Centre of Excellence, Kaunas University of Technology, LT-51368 Kaunas, Lithuania; sarunas.grigaliunas@ktu.lt (Š.G.); rasa.bruzgiene@ktu.lt (R.B.)

**Keywords:** cybersecurity, quantum-inspired security, information persistence, entropy, latency, thermodynamics of computation, Landauer’s principle, quantum Zeno effect, control lattice, security persistence index

## Abstract

This paper proposes a quantum-inspired hypothesis that cybersecurity can be modeled as information persistence: the maintenance of separation between protected and adverse system states under entropy, latency, and control cost. The objective is to provide a time- and energy-aware framework for comparing security architectures without claiming that cybersecurity is literally quantum or that a universal law has been proven. We define a dimensionless Security Persistence Index, P=Δ/(E+L+S), and map controls across three temporal phases—Intent, React, and Resolve—within a 5×3 Control Lattice. The resulting Principle of Energetic Asymmetry predicts that React-dominated architectures should require greater energy, latency, and residual-entropy cost than architectures that shift control weight toward Intent and Resolve. We evaluate this prediction through a simulation of four architectures—Intent-heavy, Balanced, Misaligned, and React-heavy—using 1000 trials per condition. The expected pattern was observed: Intent-heavy achieved the highest simulated persistence, $P_{\mathrm{sim}}=5.93$, vs. 3.45 for React-heavy, and lower normalized energy cost, CPU load, false positives, latency, and residual entropy. These results provide simulation-based internal-consistency evidence only; the framework remains a hypothesis requiring hardware-level measurement, independent replication, and field validation.

## 1. Introduction

Cybersecurity today is often implemented as a collection of tools, best practices, incident-response procedures, and regulatory controls layered onto systems after design. This operational view is useful, but incomplete. It explains how security is practiced, but not why security-like behavior recurs wherever systems must preserve identity, function, or state under disturbance. This paper therefore advances the hypothesis that security behaves as a natural-law-like persistence process: the active maintenance of separation between protected and adverse system states over time.

Recent cybersecurity research already points toward this broader interpretation. Zero Trust Architecture shifts defensive focus away from static network perimeters and toward users, assets, resources, and continuously evaluated access conditions [1]. Cyber-resilient systems engineering treats security as a life-cycle property of systems that must anticipate, withstand, recover from, and adapt to adverse conditions [2]. Cyber-resilience surveys similarly emphasize preparation, absorption, recovery, adaptation, and measurable continuity under attack or disruption [3,4]. Moving target defense research shows that deliberate structural and temporal variation can increase attacker uncertainty and reduce the advantage of static configurations [5]. These studies establish important foundations, but they generally treat resilience, access control, uncertainty, and operational response as separate engineering concerns rather than as coupled expressions of a single persistence constraint.

At its foundation, security represents the act of preserving separation between an asset and a threat over time. This separation may take spatial, temporal, logical, cryptographic, organizational, or probabilistic form, but it is always actively maintained. In physical systems, failure to maintain separation can appear as diffusion, decoherence, contamination, or collapse of an ordered state. In biological systems, it may appear as infection, mutation, immune failure, or organismal death. In digital infrastructures, it appears as compromise, corruption, privilege escalation, configuration drift, data leakage, or cascading system failure. In each case, the absence of security is not merely a policy failure. It is a transition from a preserved state toward disorder, ambiguity, or loss of functional identity.

We define this maintained separation as security. In this paper, security denotes the active maintenance of separation between a protected system state and an adverse system state over a finite time horizon. This usage is consistent with classical protection and information-flow work, where security depends on controlling access, propagation, and distinguishability among system states. A protected state is a configuration in which an asset retains its intended identity, function, authority, and informational boundaries. An adverse state is a configuration in which those properties are corrupted, confused, captured, delayed, or made indistinguishable from an unauthorized state. Separation, denoted Δ(t), is the measurable distance between these protected and adverse states. It may be logical, cryptographic, temporal, spatial, organizational, or probabilistic. Persistence is the maintenance of this separation over the interval [0,T], rather than its presence at a single audit moment. Identity preservation means that the system retains the mappings that make it itself: entity-to-attribute mappings, authority mappings, configuration baselines, state histories, and intended functional behavior. The central questions therefore become: how does security arise as a corrective process, what cost is required to maintain it, how does the timing of controls affect that cost, and how can systems be designed to preserve identity while respecting constraints imposed by information, energy, and time?

Two universal forces degrade system stability. The first is entropy, defined here in an information-theoretic and operational sense as the tendency for uncontrolled interaction with the environment to increase uncertainty, ambiguity, and state drift. Entropy manifests as unpredictability in configuration variables, identity mappings, trust relationships, and system state. In operational cybersecurity, it appears as configuration drift, identity sprawl, patch lag, stale authorization, data ambiguity, logging inconsistency, or the gradual erosion of coherence within complex infrastructures.

The second force is latency. Latency is not simply the passage of time, but the accumulated resistance that impedes movement, advancement, control action, or state transition within a system. In physical space, a snowstorm functions as a latency injector for transportation. In information systems, multi-stage authorization, deep inspection pipelines, delayed telemetry, complex change-approval procedures, or overloaded incident-response queues can function as latency injectors. Wherever resistance slows state transition, latency accumulates and creates temporal exposure windows during which adverse states can arise, propagate, or become harder to distinguish from legitimate behavior.

Both forces are measurable. They appear in thermodynamic systems, quantum information processes, networked infrastructures, cognitive bottlenecks, and organizational workflows. Security mechanisms counteract these forces through structured energy expenditure, state organization, uncertainty reduction, response-time compression, and preservation of identity. However, resisting entropy and latency imposes cost. Recent finite-time Landauer work shows that information erasure and state reset are not only bounded by ideal thermodynamic limits, but also become more costly when performed under finite-time constraints [6]. Recent work on measurement-induced Quantum Zeno dynamics likewise shows that repeated or continuous measurement can change system evolution rather than merely observe it [7]. In cybersecurity operations, the same structural pattern appears in React-heavy environments: high-volume alarms, false positives, and manual validation can overwhelm analysts and reduce effective defensive performance [8,9].

The objective of this work is to reframe security as a corrective mechanism that emerges naturally within persistent systems. By grounding security in information persistence, thermodynamic cost, latency, and temporal control placement, it becomes possible to model security as a time-dependent and energy-aware separation process. Security therefore does not arise only from fear, compliance, or adversarial conflict. It arises from necessity: any system that must continue to be itself must resist the forces that blur, delay, corrupt, or collapse its identity.

Despite the progress of recent security and resilience research, the following gaps remain. Resilience frameworks describe capabilities such as preparation, absorption, recovery, and adaptation, but they do not provide a single persistence index that relates maintained separation to energetic, entropic, and temporal burden. Cyber-risk and security-metrics research continues to report limitations in data availability, metric standardization, and cross-domain comparability [3,10]. Cyber-resilience and cyber-risk metrics motivate this work, but they do not subsume it. Resilience metrics typically evaluate whether a system can prepare for, absorb, recover from, and adapt to disruption, with emphasis on continuity of function after stress [2,3,4]. Risk metrics typically estimate likelihood, impact, exposure, or expected loss, and therefore support prioritization under uncertainty [10]. Operational security research has documented the cost of alert-heavy environments, but this cost is rarely connected to a general model, explaining why controls placed in the React phase should become more expensive than controls shifted toward preparation and closure [8,9]. What remains underdeveloped is a falsifiable model of security as information persistence. The model proposed here asks a question: over a finite time horizon, how much protected–adverse state separation is actively maintained per unit of energetic cost, latency, and residual entropy? Its unit of analysis is therefore not expected loss, control inventory, or post-disruption recovery capacity, but persistence of distinguishability over time.

The requirement for persistence can be formalized using the Security Persistence Index *P*, defined over a finite time horizon [0,T]. The purpose of this index is not to impose a single universal metric, but to provide a structured analytical template that can be instantiated with domain-specific measurements. After normalizing cost, latency, and entropy terms to a common dimensionless scale, persistence can be written as

(1) $$P\left(t\right)=\frac{\Delta (t)}{E(I,R,V)+L(t)+S(t)}$$

where

- Δ(t) represents the quantified Active Separation between asset and threat, corresponding to the degree of identity preservation within the system;
- E(I,R,V) represents the energetic cost of defensive controls distributed across three operational phases: Intent (I), React (R), and Resolve (V);
- *L* denotes latency, capturing temporal delays that create exposure windows within the system;
- *S* represents entropy, reflecting disorder or uncertainty affecting system configuration and state.

Because Δ, *E*, *L*, and *S* may be measured in different units, the index is evaluated only after normalization to a common dimensionless scale. For a raw measurement *x*, let

(2) $$\hat{x}=\mathrm{clip}\left(\frac{g\left(x\right)-g\left(x_{\min}\right)}{g\left(x_{\max}\right)-g\left(x_{\min}\right)},0,1\right),$$

where $x_{\min}$ and $x_{\max}$ are domain-specific lower and upper reference bounds, g(x)=x for approximately linear metrics, and g(x)=log(1+x) for heavy-tailed operational metrics such as alert volume or response delay. Thus *E*, *L*, and *S* represent normalized burden terms. Separation is normalized in the opposite direction: $\hat{\Delta }=0$ means no useful distinguishability between protected and adverse states, while $\hat{\Delta }=1$ means maximum attainable separation under the chosen measurement scheme.

For a concrete deployment, Δ may be estimated as a weighted combination of control objectives,

(3) $$\hat{\Delta }\left(t\right)=\sum_{m=1}^{M}\alpha_{m}d_{m}\left(t\right),\;\sum_{m=1}^{M}\alpha_{m}=1,$$

where $d_{m}\left(t\right)\in \left[0,1\right]$ measures distinguishability for objective *m*, such as valid identity binding, configuration integrity, privilege separation, recoverability, or data-flow containment. The denominator is then

(4) $$D\left(t\right)=\hat{E}\left(I,R,V\right)+\hat{L}\left(t\right)+\hat{S}\left(t\right)+\epsilon ,$$

where ϵ>0 prevents division by zero. The resulting sensitivity is transparent:

(5) $$\frac{\partial P}{\partial \Delta }=\frac{1}{D(t)},\;\frac{\partial P}{\partial E}=\frac{\partial P}{\partial L}=\frac{\partial P}{\partial S}=-\frac{\Delta (t)}{D{\left(t\right)}^{2}}.$$

Thus, the index increases linearly with maintained separation and decreases with each burden term in proportion to the current separation and total burden. In a cloud administration scenario, for example, Δ can be estimated from valid privileged-action binding and rollback assurance; *E* from CPU, memory, power, and analyst effort; *L* from policy-decision and approval delay; and *S* from residual configuration drift, unresolved alerts, and post-change ambiguity.

This formulation implies that persistence is not achieved merely by adding more controls. A system becomes more persistent when it maintains greater separation while reducing the combined burden of energy, delay, and residual uncertainty. The index therefore converts security architecture into a measurable optimization problem: maximize maintained separation while minimizing the cumulative cost of preserving that separation.

With this work, we make the following contributions:

- We define security as maintained separation between protected and adverse states over time, under the universal burdens of entropy and latency;
- The work introduces the Security Persistence Index $P_{T}$ as a time-dependent, energy-aware template for measuring information persistence;
- We organize controls into three temporal phases—Intent, React, and Resolve—and combine these phases with five analysis categories to form a 5×3 Control Lattice of fifteen phase-specific control states;
- The research derives the Principle of Energetic Asymmetry where architectures dominated by React-phase measurement should incur higher energy, latency, and residual entropy than architectures that shift control weight toward Intent and Resolve;
- We provide an initial simulation-based validation of this prediction by comparing Intent-heavy, Balanced, Misaligned, and React-heavy architectures.

The validation is intentionally framed as an initial falsifiability test rather than as final physical proof. The primary experiment compares four architectures using 1000 trials per condition and records CPU load, false-positive volume, mean time to recovery, compromise rate, residual entropy, latency, energy cost, and the simulated Persistence Index $P_{\mathrm{sim}}$. The Intent-heavy architecture achieves the highest mean simulated persistence, $P_{\mathrm{sim}}=5.93$, compared with 3.45 for the React-heavy architecture. Relative to React-heavy, Intent-heavy reduces energy cost by 52%, CPU load by 52%, false-positive volume by 71%, latency by 36%, and residual entropy by 35%. These results provide internal consistency evidence that the model behaves according to its falsifiable prediction under physically plausible assumptions, while motivating hardware-level and field validation.

The remainder of this paper is organized as follows. Section 2 reviews related work in information theory, thermodynamics of computation, quantum measurement, formal security models, cyber-physical security, cyber resilience, moving target defense, and alert fatigue. Section 3 introduces the temporal phases of Intent, React, and Resolve. Section 4 develops the energetic constraint using Landauer-style cost, Zeno-like measurement overload, coherence, and latency-resistant correlation. Section 5 formalizes the multidimensional Control Lattice and its fifteen control states. Section 6 discusses implications, critiques, and the falsifiability criterion. Section 7 applies the Control Lattice and Persistence Index to illustrative design scenarios before outlining validation pathways. Section 8 reports the primary simulation experiment, states the simulation boundaries, consolidates empirical validation pathways, and summarizes limitations and future work. Section 9 concludes by summarizing security as a physics-inspired model of information persistence.

## 2. Related Works

The conceptual basis of this manuscript lies at the intersection of information theory, thermodynamics of computation, quantum measurement, formal security models, and cyber resilience. Prior scholarship has shown that uncertainty is measurable, information processing has physical cost, repeated observation can alter system dynamics, and resilient architectures depend on how corrective mechanisms are distributed across structure and time [11,12,13,14,15,16,17,18,19,20]. However, these lines of work are usually developed in disciplinary isolation. The present article builds on them to argue that security can be interpreted as the active preservation of separation between an asset and a threat under entropy, latency, and energetic constraints.

At the informational level, the most important starting point is Shannon’s definition of entropy as a measure of uncertainty in communication systems [11]. This provides the mathematical foundation for treating security degradation as a change in uncertainty, ambiguity, and distinguishability. The physical dimension of this argument emerges from Landauer’s demonstration that logically irreversible computation has a minimum thermodynamic cost [12]. Bennett later clarified the relationship between reversibility, erasure, and Maxwell’s demon, making explicit that information manipulation is inseparable from physical dissipation [13]. Subsequent work refined these principles by tightening Landauer-type bounds [21], experimentally validating information-to-energy conversion [22], and directly confirming Landauer’s principle in controlled systems [23]. Further theoretical developments linked prediction to dissipation [24] and synthesized the thermodynamics of information as a unified field [14]. These results are directly relevant to the manuscript’s position that security operations, especially reactive ones, are never free and should be modeled as state-preserving work.

A second relevant stream comes from quantum measurement and decoherence. Misra and Sudarshan formalized the Quantum Zeno Effect, showing that frequent measurement can inhibit state evolution [15]. Experimental confirmation followed in the work of Itano et al. [25], while later analysis generalized the phenomenon into Quantum Zeno dynamics [16]. In parallel, research on decoherence established how environmental interaction destroys coherent state persistence. Joos and Zeh described the emergence of classicality through system–environment interaction [26], and Zurek synthesized decoherence, einselection, and the transition from quantum to classical behavior [17]. Schlosshauer further systematized the measurement problem and interpretations related to decoherence [27]. For the present manuscript, these studies offer a productive analogy: excessive monitoring and intervention can change the very state one seeks to preserve, while uncontrolled coupling to the environment accelerates collapse.

Work on quantum error correction is also highly relevant because it formalizes persistence under noise through structured redundancy and correction. Shor introduced one of the first practical schemes for reducing decoherence in quantum memory [28], and Steane developed error-correcting codes in quantum theory that made fault-tolerant preservation of encoded states more concrete [29]. Knight, Plenio, and Vedral explicitly linked decoherence and quantum error correction [30], while Lloyd and Slotine extended these ideas to analog quantum variables [31]. Sarovar and Milburn later described continuous quantum error correction as a cooling process that extracts entropy from the system [32], and Oreshkov and Brun examined continuous error correction under non-Markovian decoherence [33]. More recent work has shown that quantum error correction can convert coherent noise into effectively decohered noise [34] and has examined robustness-optimized correction under realistic noise assumptions [35]. This literature is important because it supports the manuscript’s claim that persistence is not passive; it depends on pre-positioned corrective structure, redundancy, and energy expenditure.

The temporal dimension of control in this manuscript also resonates with work on time symmetry and state conditioning. Aharonov, Bergmann, and Lebowitz described time symmetry in quantum measurement processes [36], and the later Two-State Vector Formalism provided a framework in which present states are constrained by both prior preparation and posterior selection [37]. Although these works arise from the foundations of quantum mechanics rather than cybersecurity, they are useful here because they motivate the distinction among controls that are embedded before interaction, triggered during interaction, and consolidated after interaction. This temporal structuring aligns with the manuscript’s Intent–React–Resolve framing.

In cybersecurity proper, the closest historical antecedents are formal theories of protection, separation, and information flow. Lampson framed protection as control over access to objects in a system [38]. Saltzer and Schroeder then articulated foundational design principles such as least privilege, fail-safe defaults, and economy of mechanism [18]. Denning’s lattice model established a mathematical theory of secure information flow [19], and Myers and Liskov later decentralized this model for distributed systems and variable trust domains [39]. These works remain central because they define security as structured separation and controlled propagation, which maps closely to the manuscript’s thesis that security is fundamentally the preservation of bounded relations over time.

The system-level and cyber-physical security literature strengthens this view by treating protection as a control problem rather than a patching exercise. Cárdenas, Amin, and Sastry identified security of control systems as a research domain where sensing, feedback, and survivability are inseparable [40]. In companion work, the same authors argued for secure control as a path toward survivable cyber-physical systems [41]. This system perspective is especially close to the present article because it emphasizes that security effectiveness depends on when and where feedback is inserted, how latency accumulates, and how system state is preserved under attack.

Another directly relevant field is resilience engineering. Sterbenz et al. surveyed resilience and survivability in communication networks and argued that persistence, graceful degradation, and recovery are architectural properties [42]. Linkov et al. proposed resilience metrics for cyber systems [20], and later work expanded resilience into a broader paradigm emphasizing preparation, absorption, recovery, and adaptation [43,44]. Woods distinguished resilience from mere robustness by stressing sustained adaptive capacity under surprise [45]. Systematic reviews of cyber-resilience assessment frameworks have since consolidated methods for evaluating such capacities across domains [3]. This body of work supports the manuscript’s insistence that security should be judged not only by prevention, but by persistence of functional coherence despite disturbance.

The adaptive defense literature contributes a further layer by examining how dynamic change can increase attacker uncertainty. Moving target defense was formalized as a strategy for creating asymmetric uncertainty for attackers through controlled variation in system properties [46]. Subsequent surveys have synthesized its mechanisms and trade-offs in network defense [5], and performance-based cyber-resilience metrics have been applied to moving target defense experimentally [47]. These studies are relevant because they operationalize the idea that security can be maintained by manipulating temporal and structural relationships rather than solely by blocking individual attack actions. In other words, the environment itself can be made persistence-preserving.

Finally, the operational burden of reactive security has been studied under the theme of alert fatigue. Recent work shows that large detection pipelines can overwhelm analysts, reduce signal quality, and degrade overall defensive performance [9,48]. This literature is significant for the present manuscript because it provides empirical support for the claim that React-heavy architectures can consume excessive energy, attention, and coordination while still failing to preserve system coherence. In that sense, alert fatigue can be interpreted as an organizational analogue of excessive measurement: the cost of observation rises while effective control weakens.

Table 1 positions the proposed Intent-heavy and React-heavy architectural projections against established cybersecurity paradigms. The purpose of the comparison is not to replace risk analysis, resilience engineering, Zero Trust, moving target defense, or alert-driven operations. Rather, it clarifies the distinct question asked by the present model: how much protected-adverse state separation is maintained over time per unit of energetic, temporal, and entropic burden. Existing paradigms remain valuable, but they usually emphasize compliance presence, risk prioritization, continuity of function, trust decisions, attacker uncertainty, or detection performance. The Persistence Index instead evaluates whether an architecture maintains separation at sustainable cost, and the Control Lattice explains why two systems with similar nominal controls may differ if one is Intent-heavy while the other is React-heavy.

Taken together, the literature supports four propositions central to this paper. First, uncertainty and state distinguishability are measurable and structurally constrained [11,49,50]. Second, preserving ordered state under noise requires redundancy, correction, and  dissipation-bearing interventions [12,14,28,32]. Third, observation and response can themselves perturb the state being protected [15,16,27]. Fourth, resilient security depends on temporal placement of controls across preparation, reaction, and recovery [5,18,40,44]. What remains insufficiently articulated in prior work is a unified model that treats security itself as a persistence law operating across physical, biological, and digital systems. The present manuscript advances that synthesis by positioning security as the active maintenance of separation over time under entropy, latency, and energy constraints.

## 3. The Temporal Phases: Intent, React, Resolve

Security is not an event. It is not a moment of defense, a snapshot of compliance, or  a single line of code. It is a temporal process, a sustained correction of drift and delay that unfolds across time. Recognizing this is essential: time is not a backdrop for security; it is a dimension in which security operates.

We propose a three-phase model of control: Intent, React, and Resolve; with each phase aligned with a distinct relationship to entropy, latency, and energetic cost. These are not stages in a life cycle, but active waveforms whose influence can echo forward and backward in time, shaping both system posture and system memory.

Let $x_{t}$ denote the security-relevant system state at time *t*, $a_{t}$ an adverse or environmental perturbation, and $u_{I},u_{R},u_{V}$ the controls assigned to Intent, React, and Resolve. A single interaction can be represented as

(6) $$x_{t}^{I}=I\left(x_{t},u_{I}\right),$$

(7) $$y_{t}=M_{R}\left(x_{t}^{I},a_{t},u_{R}\right),$$

(8) $$x_{t}^{R}=R\left(x_{t}^{I},y_{t},u_{R}\right),$$

(9) $$x_{t+1}=V\left(x_{t}^{R},h_{t},u_{V}\right),$$

where I constrains the admissible state space before interaction, $M_{R}$ measures or detects state deviation during interaction, R applies real-time containment or correction, and V verifies, records, restores, or updates the system history $h_{t}$ after interaction. The transition from Intent to React occurs when residual uncertainty or adversarial contact exceeds the precommitted boundary conditions. The transition from React to Resolve occurs when the interaction is closed and the system must prove, restore, or learn from the outcome. Resolve then feeds forward into future Intent by updating baselines, policies, evidence, and recovery assumptions. These are engineering phase transitions, not literal quantum state transitions.

### 3.1. Intent: Coherence and State Preparation

This is the phase before interaction. It precommits structure, identity, and context. Controls in this phase include classification, access design, segmentation, identity prebinding, and anticipatory configuration. In physical terms, Intent corresponds to preparing low-entropy initial conditions and boundary conditions that restrict the space of possible evolutions.

Intent is the most energy-efficient phase. Like a low-entropy initial condition in thermodynamics, it defines what “secure” means before entropy has a chance to accumulate. In quantum-inspired vocabulary, Intent is coherence: it establishes a structured, low-entropy state that can persist longer under perturbation.

### 3.2. React: The Zeno Threshold and Energetic Cost

This is the moment of contact. Detection, gating, suppression, and real-time policy enforcement, all occur here. React is necessary when the system must observe and respond under uncertainty. However, it is also the most energetically expensive phase: it consumes CPU, attention, and cognitive resources. React is the Newtonian equivalent of the Quantum Zeno Effect, constant measurement that freezes progress at the cost of performance.

This elevated cost has a structural basis in QIT terms. React is the only phase that by default maps to two or more QIT properties simultaneously: measurement and observation (the Quantum Zeno Effect cost) and state erasure or reset (Landauer’s Principle cost). Both are intrinsic to React’s function in which to respond in real time, a system must both observe and correct. Intent, by contrast, maps to a single QIT property: coherence and low-entropy state preparation. Resolve likewise maps to a single property: time-symmetric boundary conditioning in the Two-State Vector Formalism. This structural multi-mapping is why React’s energy cost is not merely higher but categorically different: it pays two independent thermodynamic costs by default, while the other phases each pay one.

In many modern security architectures, React dominates. However, overreliance here is thermodynamically unsustainable. Just as frequent quantum observation collapses superposition and increases entropy, constant real-time defense drains resources and introduces new vulnerabilities (false positives, operator fatigue, and cascading failures).

### 3.3. Resolve: Time Symmetry and Uncertainty Closure

This is the after-contact phase. Controls here include logging, forensics, non-repudiation, error correction, and rollback mechanisms. Resolve closes uncertainty loops and seeds future Intent. In the language of quantum information, Resolve plays a role reminiscent of time-symmetric formulations such as the Two-State Vector Formalism, in which both past and future boundary conditions constrain present states. Strong Resolve mechanisms tighten the coupling between outcomes and future design, reducing the need for high-frequency React. They also extend the effective coherence time of security-relevant state by enabling error detection and recovery after disturbances. In system design, it explains why verification and accountability mechanisms harden the future without needing to act in the now.

Resolve is often overlooked or minimized, but it is the bridge between history and prevention. Just as biological memory improves immune responses and quantum error correction extends coherence time, Resolve gives security a temporal backbone. It reduces the need for high-frequency React by improving both post-event clarity and future precommitment.

This temporal model is not arbitrary. It maps directly to system energy profiles, human attention patterns, and information flow:

A clarification on the “Moderate” cost assigned to Resolve in the Table 2: this cost is conditional, not intrinsic. In its baseline function of routine logging, verification, and audit, Resolve operates at low additional cost, analogous to reading an already-written record rather than erasing and rewriting one. The moderately elevated cost documented in the simulation arises specifically when Resolve is triggered as a catch for failed React: when it must close uncertainty loops that React did not close, retroactively reconstruct state, or compensate for incomplete containment. In this mode, Resolve inherits a portion of React’s entropic burden. A clean Intent to Resolve path, one that minimizes or bypasses React, carries significantly lower Resolve cost than the table’s “Moderate” label implies in isolation.

Security, in this framing, becomes a time-aware optimization problem: how to minimize disorder and delay while conserving energy and preserving state. Systems designed with strong Intent and Resolve can shrink or even eliminate portions of React, like a well-prepared quantum system that needs no measurement mid-state to ensure fidelity. Ultimately, this model allows us to ask the most important design question:

How much of your security is trapped in React, and what would it take to shift it toward Intent and Resolve?

## 4. The Energetic Constraint

If entropy and latency are universal forces, then any system that seeks to persist must develop mechanisms to resist them. In physics, this is not hypothetical, it is formalized. In quantum information theory (QIT) and thermodynamics, the preservation of state under uncertainty is not only measurable, it is mathematically constrained. We argue that the behaviors required for security already exist in these domains, and that security itself is a macroscopic expression of their principles.

The energetic constraint is treated here as a model of operational cost, not as a claim that cybersecurity controls literally instantiate quantum dynamics. The mechanism in deployed systems is ordinary computation, communication, storage, coordination, and human decision effort. The physics-inspired claim is narrower: information-preserving operations such as measurement, correction, reset, rollback, and verification are not free, and their cost should depend on when they are performed and how much uncertainty they must resolve.

### 4.1. The Landauer Boundary: Minimum Energetic Cost

Landauer’s Principle formalizes a hard truth: erasing information costs energy. Specifically, resetting a single bit incurs a minimum cost of *kT ln* 2 (where *k* is Boltzmann’s constant and *T* is temperature). In security, this principle explains why reactive remediation, rollback, reauthentication, and re-encryption are the most expensive form of control.

Prevention is not just better than cure, it is thermodynamically cheaper.

### 4.2. The Zeno Effect: Exponential Cost of Measurement

In quantum physics, observation is not free. Measuring a system collapses its probabilistic waveform into a fixed state, and that collapse introduces cost: energetic, informational, and temporal. This is captured in the Quantum Zeno Effect: frequent observation slows evolution, freezes progress, and amplifies energetic cost.

In security, this is React. Constant monitoring, polling, and active detection all consume resources, attention, CPU cycles, bandwidth, and increase the likelihood of error or failure under stress. Monitoring is commonplace, but without coherence (Intent) and post-verification (Resolve), it becomes a denial-of-service on your own system. Security design must treat monitoring like measurement, as a scarce resource, not a default control.

### 4.3. Coherence: Resistance to Entropy and Drift

In quantum systems, coherence refers to the stability of a quantum state over time and the ability to maintain superposition without collapsing into noise. Loss of coherence, or decoherence, represents entropy: uncontrolled interaction with the environment that degrades structure and predictability.

Security faces the same challenge. A coherent system, whether digital, biological, or organizational, requires mechanisms to sustain its identity against disorder. Configuration drift, bit rot, policy decay, and insider threat all manifest as forms of decoherence. Security measures, like hashing, checksums, version control, and attestation, are coherence-preserving endeavors. Their goal is to extend the lifetime of meaningful state in the face of constant noise.

### 4.4. Superposition and Entanglement: Defining System Separation

Superposition is the quantum principle that a system exists in multiple possible states simultaneously until observed. This probabilistic encoding is more efficient than classical determinism, as it stores ambiguity without collapse.

Security, too, must operate under uncertainty. It must encode intent, verify claims, and decide access before full certainty is available. Restrictive architectures, probabilistic authentication, and anomaly detection all operate like quantum superposition: they evaluate possible states before making irreversible commitments. The closer a system models uncertainty natively, the less energy it spends correcting poor assumptions later.

### 4.5. The Two-State Vector Formalism: Time as a Control Surface

Traditional physics views state as evolving from the past, but the Two-State Vector Formalism in the language of quantum information posits that a system’s present is shaped by both past and future boundary conditions. This is not mere theory, it has predictive power in quantum experiments.

Security mirrors this logic. Strong post-event controls (Resolve) shape future behavior and reduces React cost. Conversely, prebound intent (e.g., secure boot, path restrictions, pre-signed claims, immutable logging) makes real-time measurement less necessary. Security behaves like a bidirectional waveform, Intent and Resolve co-shaping the present, minimizing the need for mid-state React.

These correspondences are used as functional mappings rather than literal quantum identities. Coherence is used to model policy persistence; entanglement is used to model distributed trust; the Zeno Effect is used to model vigilance fatigue; and Landauer-style cost is used to model the computational and human cost of rework.

Security, viewed through this lens, is not an abstract design goal. It is information preservation under universal physical constraints. This alignment gives us not only explanatory power, but predictive power, and a basis for building systems that do not just defend but endure.

### 4.6. Entanglement: Latency-Resistant Correlation

Quantum entanglement allows correlated behavior across distance without direct communication: what affects one particle instantaneously affects its partner. This is not a violation of causality, but a redefinition of correlation: identity is distributed across space and time.

Security systems increasingly rely on entangled design: distributed trust, federated identity, secure multi-party computation, and consensus mechanisms which all require state correlation across components. Entanglement in this context is a design goal, systems that do not rely on synchronous React, but instead share context robustly enough that future actions can be inferred, authorized, or verified without active polling.

Entanglement shows us that latency can be designed around, not just reacted to.

## 5. A Multidimensional Model

If security is a physical process that resists entropy and latency, then the mechanisms by which it does so must be structured, quantifiable, and predictable. To move beyond ad hoc practices, we must define controls not as standalone techniques, but as states in a system, precise configurations with observable effects under stress. In this section, we introduce a formal model in which security controls behave as eigenstates within a multidimensional attribute space.

### 5.1. Controls as Eigenstates in Hilbert Space

In quantum mechanics, an eigenstate is a pure, stable configuration of a system that, when measured, yields a predictable result (its eigenvalue). These states form an orthogonal basis in Hilbert space, a mathematical space where complex interactions can be modeled as linear combinations of basis vectors.

We model security controls as vectors in a multidimensional Hilbert space, where the system’s overall security state is a superposition of these control eigenstates. This structure is defined by the Control Lattice, which is organized by two orthogonal axes, ensuring non-overlapping measurement and analysis:

1. Temporal Alignment (Phase)—the three functional phases of security: Intent (I), React (R), and Resolve (V).
2. Analysis Category (5-Point Process)—the five core categories used for thorough analysis of any asset or interaction (see Appendix A for a concise description of the OSSTMM Five-Point Process).

**Figure 1:**
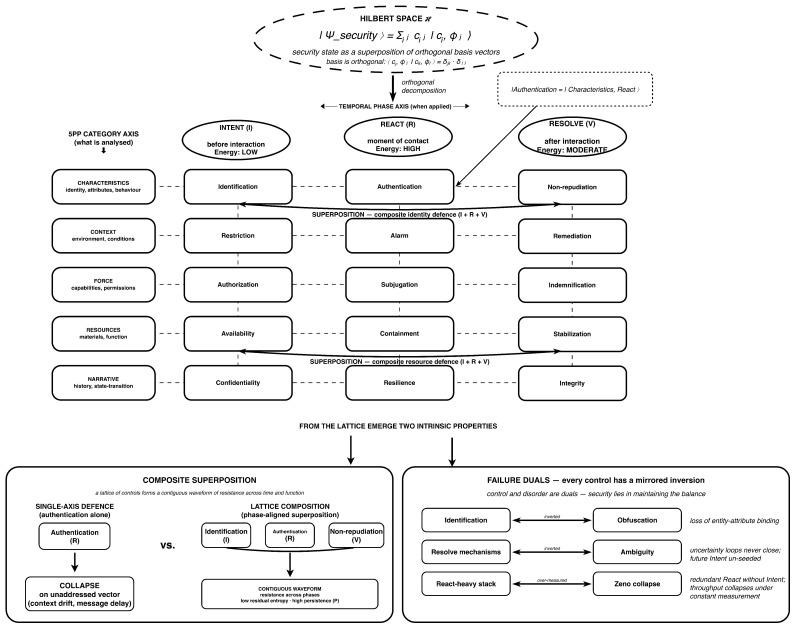
Conceptual structure of the multidimensional model.

Each ordered pair (category, phase) corresponds to a distinct control eigenstate. This yields 15 core controls, which can be arranged in a 5×3 matrix (Table 3):

These labels are intentionally broad. For example, “Identification” in the Intent/ Characteristics cell refers to any mechanism that establishes and maintains mappings between entities and attributes before interaction; “Containment” in the React/Resources cell covers controls that limit the blast radius of resource misuse during an incident; and “Integrity” in the Resolve/Narrative cell captures mechanisms that ensure histories and records remain trustworthy over time. The orthogonality of these two axes is paramount: a control’s function is defined by what it is analyzing (e.g., Resources) and when it is applied (e.g., Intent), allowing for a clean, linear combination of control vectors that mirror the basis states of Hilbert space. Figure 1 renders the multidimensional model as a conceptual diagram rather than as a matrix.

The Hilbert space H sits at the top of the figure with the decomposition $|\Psi_{\mathrm{security}}\rangle =\sum_{ij}c_{ij}\;|c_{i},\varphi_{j}\rangle$ written explicitly inside it, together with the orthogonality condition $\langle c_{i},\varphi_{j}|c_{k},\varphi_{\ell }\rangle =\delta_{ik}\;\delta_{j\ell }$. An orthogonal-decomposition channel beneath the Hilbert cloud flows into the lattice region, making the move from abstract state to concrete basis visually explicit. The three phase ellipses across the upper register carry the temporal basis and are color-coded by their characteristic energetic cost, with Intent the lowest, React the highest, and Resolve at an intermediate level. The five category cards along the left margin form the 5-Point-Process basis and remain neutral with respect to time. Each of the fifteen control eigenstates is then identifiable both by its position in the lattice and by the color it inherits from its phase column, while the dashed phase and category rails extend the basis structure across the entire analysis surface so that every eigenstate appears at the intersection of two visible projections. A side callout picks out one node, Authentication, and labels it as |Characteristics,React〉 to fix the eigenstate notation against a concrete control. Each control thus becomes a unique combination in a high-dimensional vector space, akin to a quantum eigenstate. When applied (i.e.,“measured” under attack or stress), the system yields a deterministic behavior: block, allow, redirect, degrade gracefully, etc. These outcomes are the eigenvalues, predictable system responses tied to control placement.

### 5.2. The Control Lattice: 15 States of Resistance

The current research defines a 120-cell matrix, derived from attributes, states of activity, time phases, and spanning types of processes (such as trust, privacy, attack, and security). These lead us to 15 states of resistance, better known as security controls, which are not arbitrary as they represent the full set of separable, orthogonal interventions that can be applied to maintain separation between an asset and a threat. Each is designed to resist entropy or latency. For example, in the Security Control Lattice:

- Identification is the control located at the intersection of the Intent Phase and the Characteristics Category;
- Containment is the control located at the intersection of the React Phase and the Resources Category.
- Integrity is the control located at the intersection of the Resolve Phase and the Narrative Category.

The combinatorial nature of the lattice ensures that every control has a mirrored failure mode: what happens when a control is denied, misplaced, misconfigured, or inverted. Identification’s inversion becomes Obfuscation; Resolve’s failure leads to Ambiguity. This gives the model a symmetry that mirrors physical systems: control and disorder are duals, and security lies in maintaining the balance.

### 5.3. Composite Interactions: Superposition and Entropy Reduction

Just as quantum systems combine eigenstates through superposition to create richer behaviors, controls in this model interact to form composite defenses. A system that uses only one control axis, say, authentication alone, may collapse when entropy hits an unaddressed vector (like context drift or message delay). However, a lattice of controls forms a contiguous waveform of resistance across time and function.

This also allows for simulation. In a simulated attack environment, one can model drift by increasing entropy in a given dimension (e.g., configuration divergence) and observe how different control combinations reduce or amplify disorder. These mappings allow us to quantify control efficacy not just by presence, but by placement and alignment.

### 5.4. Energy and Measurement: Why Control Placement Matters

Each control placement has an energy profile, just as each quantum operation has a cost. Controls placed in React (e.g., real-time blocking) consume the most energy, attention, and bandwidth. Controls in Intent (e.g., pre-approval, segmentation) are lowest-cost but require strong system design. Controls in Resolve (e.g., immutable logging, cryptographic receipts) enable retroactive validation, shifting cost from the moment of crisis to the margin of audit.

Poorly placed controls, such as redundant React checks without corresponding Intent, create Zeno-like inefficiencies, collapsing system throughput under the weight of constant measurement. Properly composed controls act as low-entropy eigenstates, preserving order across time with minimal intervention.

This eigenstate model is not an abstraction, it is a blueprint. It allows us to:

- Design controls with predictable behaviors under attack;
- Model and simulate entropy-resistance and latency-compression;
- Calculate the energetic and cognitive cost of each control placement;
- Predict failure modes when control vectors are misaligned or absent.

Security, in this framework, becomes a field theory of information preservation, where each control is a field alignment that shapes the system’s resistance curve. Just as physics describes the shape of matter through quantum states, we describe the shape of security through composable, measurable control states.

## 6. Implications and Critiques

Re-framing security as a natural law is not merely a shift in language, it is a redesign of assumptions. It changes how we measure control, how we engineer systems, and how we understand failure. If this hypothesis holds, then the implications are broad, affecting everything from how we budget cybersecurity resources to how we design AI, governance, and trust architectures.

### 6.1. The Falsifiability Criterion

For this hypothesis to transition from theory to science, the Security Persistence Index (*P*) must be falsifiable. The core testable assertion derived from Landauer’s Principle and the Control Lattice model is the Principle of Energetic Asymmetry:

The total energetic cost of persistence ($E_{cost}$) is dominated by the React phase ($E_{R}$), and a system’s persistence (*P*) is maximized by minimizing its $E_{R}$ vector projection. This requires establishing direct, measurable mappings for the variables in the core function (see Equation (1)).

#### 6.1.1. Testable Benchmarks: Mapping Abstract Physics to Measurable Data

We propose benchmarks that allow for the direct comparison of system architectures (Intent-heavy vs. React-heavy) across the core burdens:

1. Energetic Cost (*E*). *E* must be quantified via low-level consumption metrics across the three phases. Metric: total System Energetic Cost ($E_{Total}$) measured in processor cycles (CPU/GPU load), memory bandwidth consumed, and instantaneous power draw during security operations. Prediction (P1) is for any two systems achieving the same separation (Δ), the system with a greater average control vector projection onto the Intent phase will exhibit a lower $E_{Total}$ consumption over time than the system projected onto the React phase. Prediction (P2) is the relationship $E_{R}\approx L\;\cdot \;2^{\mu }$ that will be confirmed by showing that $E_{R}$ scales nonlinearly (exponentially) with the Uncertainty (μ), the bits of system state that must be measured, resolved, and corrected within the limited time window.
2. Latency (*L*) and Entropy (*S*). These variables quantify disorder and delay, the  two resistive forces of the natural law. Latency (*L*)—measured as the aggregate frictional impedance on state-transition: the sum of all factors (structural, procedural, computational, or environmental) that slow or block an entity’s movement through the system. This includes authorization chain complexity, detection pipeline depth, response process friction, and environmental resistance. Operationally quantified as the compound delay from state-change event to effective control action (e.g., Alarm or Containment). High latency increases the window of exposure, decreasing *P*. System Entropy (*S*)—defined as the measured rate of drift away from the defined, low-uncertainty Intent state (e.g., rate of configuration deviation, mean-time-to-inaccuracy of system characteristics). High *S* necessitates high-cost $E_{R}$ to correct.

#### 6.1.2. Persistence Index Comparison

The definitive experiment is to compare two systems, one designed for Maximum Intent Projection (high $E_{I}$, low $E_{R}$) and one designed for Maximum React Projection (high $E_{R}$, low $E_{I}$) against a common set of threat vectors and environmental noise.

Hypothesis. The Intent-Projected system will demonstrate a significantly higher, more stable Security Persistence Index (*P*) than the React-Projected system, proving that resistance is thermodynamically more efficient than correction.

### 6.2. From Checklist to Constraint Field

If security is entropy and latency resistance, then every control must be evaluated not by compliance to best practices, but by its measurable effect on disorder and delay. This re-frames security architecture as a constraint optimization problem. Instead of asking “Do we have MFA?” we ask “How much entropy or latency does this control correct, at what cost, and in what phase?”

This leads to radically different outcomes. Systems shift toward Intent-based architectures, where identity and authorization are precommitted rather than verified in real time. Resolve mechanisms become critical infrastructure, not audit afterthoughts. React is redesigned as an emergency-only control surface, not the default defense layer. Alert fatigue and monitoring collapse are explained as Zeno-like measurement overload, not staffing failures. Security becomes not a stack, but a field, an energetic and informational geometry defined by control alignment across time.

### 6.3. Operational Implications: Burnout as a Physics Problem

If real-time security is thermodynamically expensive, then the burnout of SOC teams and the fragility of alert-driven systems are not cultural problems, they are entropy overloads caused by poor phase alignment. A system that relies too heavily on React without Intent or Resolve is like a quantum system measured constantly until it decoheres.

This offers a measurable path to operational reform: reduce React dependency by investing in Intent and Resolve. Just as quantum systems extend coherence time through entangled preparation and error-correcting codes, security teams can regain resilience by restructuring around lower-energy phases.

### 6.4. Scientific Implications: Falsifiability and Measurement

For a theory to be scientific, it must be falsifiable. This framework makes several testable predictions. Moving controls from React to Intent or Resolve will reduce CPU usage, alert volume, and mean time to recovery without increasing breach rate. Systems with only React-phase controls will exhibit superlinear cost curves as entropy or load increases, mirroring the Zeno threshold. In simulation, controls placed as eigenstates in a Hilbert-like space will reduce drift, or entropy, more efficiently than randomly placed controls. These are not vague ideas; they are experiments waiting to be run. Simulations using entropy injection, such as configuration noise, or latency amplification, such as delayed inputs, can be used to benchmark control efficacy using standard metrics including energy, time, error rate, and coherence duration.

### 6.5. Philosophical Implications: Security Without Enemies

Traditional security models assume an attacker. However, entropy and latency do not require malice. They are natural consequences of complexity, time, and scale. This framework allows us to design security without fear, not as an adversarial posture, but as a structural requirement for persistence. This is essential in domains like AI alignment, where collapse need not be malicious to be catastrophic; decentralized governance, where trust failure arises from drift rather than treachery; space systems, where latency is physical and cannot be eliminated; and ecological modeling, where resilience depends on entropy regulation rather than perimeter defense. Security becomes less about defeating an enemy and more about maintaining identity across time in an unstable universe.

### 6.6. Critiques and Boundaries

The framework remains a falsifiable hypothesis, not a demonstrated universal law. The phrase “natural-law-like” is used in a limited sense: it denotes a recurring constraint on systems that must preserve identity under entropy, latency, and energetic cost. It does not mean that cybersecurity is reducible to quantum mechanics or that a single physical law fully explains social, organizational, adversarial, or economic dimensions of security.

Several boundaries follow. The quantum vocabulary is analogical and formal, not literal. Terms such as eigenstate, coherence, measurement, and Zeno-like overload are used to structure the model and generate testable predictions, not to claim that operational security systems occupy physical quantum states. Landauer-style energetic arguments provide lower-bound and directionality intuitions for information reset and rework; they do not directly predict the full energy consumption of hardware, software stacks, or human organizations. The present validation is simulation-based and does not yet establish external validity across deployed systems. The model abstracts from adversarial adaptation, usability constraints, institutional incentives, supply-chain dependencies, and heterogeneous infrastructure.

Alternative explanations must also be considered. An Intent-heavy architecture may outperform a React-heavy architecture because it reflects ordinary prevention-over-detection engineering, because it reduces complexity, because it improves control alignment, or because the simulation parameters favor lower synchronous cost. These alternatives do not invalidate the model, but they define what empirical testing must separate. The hypothesis would be weakened if React-heavy systems did not show increasing marginal cost under monitoring load, if entropy and latency did not correlate with persistence loss, or if field systems with similar control coverage showed no relationship between temporal control placement and *P*.

The philosophical implication is therefore modest. Security may be interpreted as an emergent persistence requirement across domains, but the scientific contribution of this paper is the narrower claim that persistence can be modeled, measured, and potentially falsified through Δ, *E*, *L*, and *S*.

## 7. Applied Design Scenarios

The Control Lattice and the Security Persistence Index are intended to guide both architecture design and empirical testing. In design use, the lattice asks where each control operates in time—Intent, React, or Resolve—and which system property it acts upon: Characteristics, Context, Force, Resources, or Narrative. The Persistence Index then asks whether a proposed architecture maintains greater asset–threat separation, Δ, at lower combined burden from energy, latency, and residual entropy. The following illustrative scenarios show how the model can be used as a design-screening tool before the later subsections describe validation experiments.

### 7.1. Using the Lattice and Persistence Index as a Design Tool

A lattice-guided design process proceeds in three steps. First, controls are mapped to cells of the Control Lattice, $\langle c_{i},\phi_{j}\rangle$, where $c_{i}$ denotes the analysis category and $\phi_{j}\in \left\{I,R,V\right\}$ denotes the temporal phase. Second, the architect identifies phase imbalance: excessive React projection indicates dependence on real-time measurement, while weak Intent or Resolve projection indicates that the system has not precommitted enough structure or closed enough uncertainty after interaction. Third, candidate designs are compared with a normalized screening version of the Persistence Index,

$$P_{\mathrm{screen}}=\frac{\Delta_{\mathrm{est}}}{E_{\mathrm{est}}+L_{\mathrm{est}}+S_{\mathrm{est}}}.$$

Here, $\Delta_{\mathrm{est}}$ is an estimated separation score, $E_{\mathrm{est}}$ is estimated operational cost, $L_{\mathrm{est}}$ is estimated latency introduced by the control path, and $S_{\mathrm{est}}$ is estimated residual uncertainty after controls execute. These values are not presented as empirical measurements; they are normalized design-screening estimates used to compare alternative architectures before implementation or instrumentation.

### 7.2. Illustrative Scenario 1: Privileged Cloud Administration

Consider a cloud service in which privileged production changes require repeated authentication prompts, manual security review, and high-volume alert monitoring. In lattice terms, the baseline architecture is concentrated in 〈Characteristics,R〉 and 〈Context,R〉: the system keeps measuring identity and context during interaction. This increases synchronous latency and analyst burden while leaving several Intent and Resolve cells underused.

A lattice-guided redesign shifts part of the control weight away from React. In the Intent phase, hardware-bound administrator identity, device posture requirements, just-in-time privilege scoping, change-window restrictions, and production segmentation precommit the allowed state space. These controls occupy 〈Characteristics,I〉, 〈Force,I〉, 〈Context,I〉, and 〈Resources,I〉. In the Resolve phase, cryptographic administrative receipts, immutable change logs, automated rollback records, and post-change integrity verification occupy 〈Characteristics,V〉, 〈Narrative,V〉, and 〈Resources,V〉. React remains present, but is narrowed to anomaly confirmation and emergency containment rather than continuous revalidation of every ordinary action.

The design implication is that the same nominal policy can be implemented with a different temporal profile. A React-heavy design asks, “Is this action safe now?” at many points during interaction. The lattice-guided design asks, “Can the system be prepared so that only a smaller set of actions requires real-time judgment, and can the result be proven and reversed afterward?” For example, using illustrative normalized screening values,

$$P_{\mathrm{baseline}}=\frac{0.70}{0.44+0.32+0.25}=0.69,$$

where the denominator reflects high real-time cost, approval delay, and residual ambiguity. After shifting controls toward Intent and Resolve,

$$P_{\mathrm{redesign}}=\frac{0.82}{0.23+0.14+0.13}=1.64.$$

The exact numbers are placeholders for design comparison, not empirical claims. The important result is the direction of the redesign: increase maintained separation while reducing the combined burden of energy, latency, and residual uncertainty.

### 7.3. Illustrative Scenario 2: Ransomware-Resilient File Service

A second scenario is a file service defended mainly through endpoint detection, alert triage, and manual containment after encryption behavior is detected. In the Control Lattice, this design is dominated by 〈Resources,R〉 and 〈Context,R〉: the system waits for suspicious resource use or abnormal context before acting. This can preserve some separation, but only after latency has already created an exposure window.

A lattice-guided redesign first strengthens Intent. Immutable backup policy, least-privilege write paths, signed update channels, segmentation between user shares and backup stores, and preapproved recovery identities reduce the reachable attack surface before contact. These controls map to 〈Resources,I〉, 〈Force,I〉, 〈Narrative,I〉, and 〈Characteristics,I〉. React controls remain useful for canary-file triggers, abnormal write-rate detection, and containment, but their role is narrowed. Resolve is then strengthened through immutable snapshots, tested restoration procedures, integrity verification, and non-repudiable recovery records, occupying 〈Resources,V〉, 〈Narrative,V〉, and 〈Characteristics,V〉.

In screening form, the baseline ransomware design may be represented as

$$P_{\mathrm{baseline}}=\frac{0.66}{0.51+0.38+0.30}=0.55,$$

because real-time detection and manual containment carry high operational cost, high latency, and substantial residual uncertainty. A redesigned service with stronger Intent and Resolve may be represented as

$$P_{\mathrm{redesign}}=\frac{0.86}{0.28+0.17+0.16}=1.41.$$

Again, the values are illustrative. Their purpose is to show how the lattice changes the design conversation. The question is no longer only whether ransomware controls exist, but whether they are placed early enough to restrict possible damage, late enough to prove and restore state, and sparingly enough in React to avoid unnecessary measurement cost.

### 7.4. Benchmarking Control Placement Across Time Phases

Hypothesis. Systems that shift controls from React to Intent and Resolve will consume less energy (CPU, bandwidth, operator time) for equivalent or better security outcomes.

Method. Implement comparable systems with equivalent policies distributed differently across the Intent–React–Resolve spectrum. Measure CPU load, false positives, recovery time, and operator burden. Simulation condition note: The existing simulation runs measured Resolve cost specifically in the condition where Resolve was triggered by failed React, that is, Resolve operating as a catch for uncertainty loops React did not close. This is one valid experimental condition but not the only one. An additional condition isolating a clean Intent to Resolve path (without React triggering Resolve) is needed to empirically establish Resolve’s baseline cost independent of React failure. Until that run exists, the claim that baseline Resolve cost is lower than the “Moderate” shown in the phase table rests on theoretical derivation from TSVF and Landauer rather than direct simulation evidence.

Expected Result. React-heavy systems will exhibit higher entropy (e.g., configuration drift, delayed resolution) and higher energy cost under stress.

### 7.5. Simulation of Zeno Thresholds in Reactive Systems

Hypothesis. Excessive real-time measurement (React) results in superlinear energy cost and increased failure rates, mirroring the Quantum Zeno Effect.

Method. Simulate a system under increasing monitoring frequency. Introduce threats, measure state resets, false positives, missed events, and system saturation points.

Expected Result. Identify a Zeno threshold where increased vigilance produces diminishing or negative returns.

### 7.6. Entropy Injection to Test Control Efficacy

Hypothesis. Controls modeled as aligned eigen-states will reduce entropy more effectively than randomly placed or misaligned controls.

Method. Simulate systems under entropy injection (e.g., random configuration drift, inconsistent identity propagation). Apply control sets with different attribute alignments. Measure order restoration rate, error propagation, and recovery efficiency.

Expected Result. Phase-aligned controls exhibit lower entropy metrics (drift, ambiguity, instability) under equivalent load.

### 7.7. Cost Modeling Using Landauer’s Bound

Hypothesis. Reactive control mechanisms that erase or reset state will correlate with higher thermodynamic and operational cost.

Method. Calculate theoretical energy cost using Landauer’s limit and compare to empirical CPU and power usage of React-heavy controls. Repeat for systems that leverage Intent or Resolve mechanisms (e.g., prebound tokens, immutable logs).

Expected Result. React controls incur measurable energetic cost consistent with or exceeding Landauer’s bound.

### 7.8. Analysis of Real-World Breaches Using the Entropy-Latency Model

Hypothesis. Breaches correlate with entropy buildup (e.g., misconfiguration) and latency gaps (e.g., delayed patching or incident response).

Method. Conduct forensic analysis of known breaches (e.g., SolarWinds, Colonial Pipeline). Map contributing factors to entropy and latency categories. Evaluate presence/absence of Intent and Resolve controls.

Expected Result. Breach severity and detection delay correlate with lack of proactive structure and weak Resolve mechanisms.

These experiments provide a foundation for empirical inquiry. If security is indeed a physical phenomenon, then its behaviors should obey measurable constraints, and its failures should be predictable by the physics of disorder, delay, and recovery.

## 8. Simulation-Based Validation and Empirical Validation Pathways

The hypothesis advanced in this paper makes a specific structural prediction: under the Persistence Index P=Δ/(E+L+S), architectures whose control weight is concentrated on the Intent phase of the temporal axis should achieve higher *P* at lower *E* than architectures whose weight is concentrated on React. This section reports a primary experiment that tests that prediction end-to-end, and registers four alternative validation pathways that probe complementary aspects of the same hypothesis.

### 8.1. Primary Validation: Architecture Comparison

#### 8.1.1. Hypothesis and Predictions

The Principle of Energetic Asymmetry yields two ordinal predictions that are directly testable in simulation. First, with total control weight held constant, $P_{\mathrm{Intent}}>P_{\mathrm{Balanced}}>P_{\mathrm{React}}$. Second, the energy budget required to reach a fixed asset-threat separation Δ is monotone increasing in the React weight, and superlinear above a stress threshold (Zeno regime). The direction of the inequalities is the falsifiable claim; failure to observe the ordering at statistical significance would disconfirm the hypothesis.

#### 8.1.2. Method

Four architectures were constructed by distributing a fixed total of phase weight $w_{I}+w_{R}+w_{V}=1$ across the three phases of the temporal axis. The Intent-heavy condition placed 70% of weight on Intent; the React-heavy condition placed 70% on React; the Balanced condition split weight evenly; the Misaligned condition redirected 35% of weight to an axis with zero coverage on the threat, modeling the operational reality of compliance spending addressed to the wrong vector. Phase-level constants were fixed before the experiment ran. Per-phase energy cost was set with React the most expensive (motivated by the Landauer bound on continuous measurement and erasure), Resolve moderate (immutable ledger maintenance), and Intent cheapest (design-time precommitment). Per-phase entropy reduction efficacy was set with prevention dominating correction and correction dominating post hoc resolution. Per-phase latency contribution was set with React introducing the dominant synchronous wall-clock cost. The React phase received a superlinear cost term proportional to $w_{R}^{2}\sigma$ in the threat-stress factor σ, implementing the Zeno overload prediction of Section 5.4.

One thousand independent trials were run per architecture from seed 20260511, yielding four thousand trials in total. Each trial sampled an entropy load and stress factor from a Gaussian process, applied the architecture, and recorded eight metrics such us CPU load, false-positive volume, mean time to recovery, compromise rate, residual entropy, latency, energy cost, and the Persistence Index $P_{\mathrm{sim}}$. Pairwise architecture differences were evaluated with Welch’s *t*-test for unequal variances.

#### 8.1.3. Reproducibility

The simulation is implemented in approximately three hundred lines of Python 3.12.8 with no external data dependencies. The script, the random seed, the raw per-trial measurements, the aggregated summary, and the figure-generation logic are all released alongside this paper. Re-running with the published seed reproduces every reported value to within floating-point precision. Source: validation_simulation.py.

#### 8.1.4. Results

**Figure 2:**
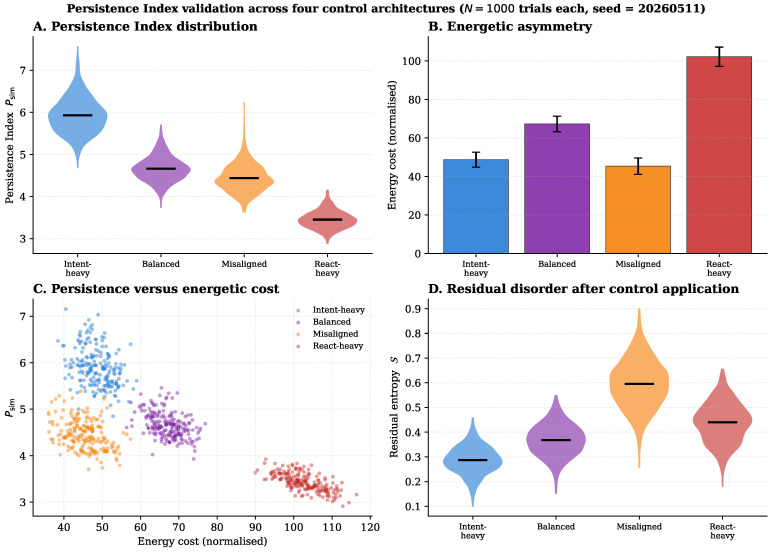
Simulation-based validation of the Security Persistence Index across four control architectures. The experiment compares Intent-heavy, Balanced, Misaligned, and React-heavy architectures over 1000 trials per condition using seed 20260511. Panel (**A**) shows the distribution of the simulated Persistence Index, $P_{\mathrm{sim}}$, for each architecture, with black horizontal markers indicating group means. The Intent-heavy architecture exhibits the highest mean persistence, followed by Balanced, Misaligned, and React-heavy architectures. Panel (**B**) reports normalized energetic cost with one standard deviation, showing that React-heavy control placement imposes the largest energetic burden, whereas Intent-heavy placement achieves higher persistence at lower cost. Panel (**C**) plots $P_{\mathrm{sim}}$ against normalized energy cost for sampled trials, demonstrating the separation between architecture classes and the negative relationship between React-dominated cost and persistence. Panel (**D**) shows residual entropy after control application; the Misaligned architecture exhibits the highest residual entropy, indicating that control alignment is a distinct predictor of persistence independent of total energy expenditure.

The numerical values reported in Table 4 and plotted in Figure 2 are generated outputs of the simulation, not assumed result values. Each architecture *A* is defined by a phase-weight vector $w_{A}=\left(w_{I},w_{R},w_{V}\right)$, where $w_{I}+w_{R}+w_{V}=1$, and, where applicable, by a misalignment term $m_{A}$ representing control weight placed on a zero-coverage axis. The experiment uses normalized simulation units rather than direct hardware joules so that the first test evaluates the ordinal prediction of the hypothesis under controlled conditions. The parameterization used in the reported run is summarized in Table 4.

The Intent-heavy architecture achieved the highest mean Persistence Index ($P_{\mathrm{sim}}=5.93$), followed by Balanced (4.66), Misaligned (4.44), and React-heavy (3.45). The ordering is consistent with the hypothesis on every metric. All pairwise differences between Intent-heavy and React-heavy were significant under Welch’s *t*-test with *p*-values numerically zero at double precision and *t*-statistics exceeding 100 in absolute value on every metric. Relative to React-heavy, the Intent-heavy condition reduced energy cost by 52%, CPU load by 52%, false-positive volume by 71%, latency by 36%, and residual entropy by 35%, while improving $P_{\mathrm{sim}}$ by 72%.

Figure 2 provides visual evidence for the Principle of Energetic Asymmetry. The Intent-heavy architecture achieves the highest mean $P_{\mathrm{sim}}$ while maintaining the lowest or near-lowest energetic burden, supporting the prediction that precommitted controls reduce the need for costly real-time measurement. By contrast, the React-heavy architecture occupies the high-cost, low-persistence region of the energy–persistence plane, consistent with the model’s claim that excessive React-phase projection increases energetic and temporal burden. The Misaligned condition is especially important because it does not simply behave as a weaker Balanced architecture; instead, it produces the highest residual entropy, showing that misplaced controls can fail even when they consume a nontrivial control budget. The figure therefore supports two claims: phase distribution affects energetic efficiency, and phase alignment with the relevant threat vector is necessary for persistence.

#### 8.1.5. Interpretation

The hypothesis predicts both a direction and a mechanism: Intent-heavy architectures should dominate not because they do more security work but because they remove uncertainty before it has to be measured. The simulation supports both predictions. The Intent-heavy condition achieved the highest Persistence Index at the lowest energy cost, and the gap widened with the stress factor as the superlinear React term began to dominate the budget. The Misaligned condition is the most informative secondary finding in that it consumed a moderate energy budget yet produced the highest residual entropy of any architecture tested, second-lowest $P_{\mathrm{sim}}$, and the highest compromise rate. Misalignment is therefore not a mild form of inefficiency. It is a distinct failure mode in which the budget is spent on an axis that the threat does not lie on, and the shortfall in coverage cannot be recovered by adding more weight on the same axis. The implication for practice is direct to defense posture, which cannot be evaluated by total spend or by phase distribution alone, only by phase distribution conditioned on threat coverage.

#### 8.1.6. Bounds

The simulation tests the structural prediction of the hypothesis, not the absolute numerical claim. Phase-level constants were motivated by physical principles (Landauer’s bound, Zeno overload) but were not measured against real hardware. The simulation should therefore be read as internal consistency evidence that the model behaves as the hypothesis requires when its parameters take physically plausible values. The four pathways below specify how a hardware-level validation, an independent replication, and two distinct cross-checks of the prediction can be carried out.

### 8.2. Alternative Validation Pathways

The Energetic Asymmetry Principle admits independent empirical tests beyond the architecture comparison above. The following four pathways are stated with sufficient method detail that any of them could be executed as a standalone falsification experiment by an independent group, and each probes a distinct aspect of the hypothesis. The pathways are ordered from cheapest to most demanding in terms of instrumentation and data access.

#### 8.2.1. Zeno-Threshold Sweep in Reactive Systems

Hypothesis. Increasing the monitoring frequency of a React-heavy system produces superlinear cost growth and a measurable threshold beyond which additional measurement worsens the metric it is intended to improve.

Method. Hold the architecture fixed at React-heavy and sweep the monitoring frequency from one to twelve in unit steps. Measure CPU load, false positives, missed events, compromise rate, latency, energy cost, and $P_{\mathrm{sim}}$ at each frequency over one thousand trials per setting. Fit linear and quadratic models to the energy curve.

Expected result. A monotonic threshold at which missed events stop improving while energy continues to grow, empirically locating the Zeno point predicted by Section 5.4. The quadratic model should outperform the linear model on $R^{2}$ by a measurable margin.

#### 8.2.2. Entropy Injection at Equal Phase Weight

Hypothesis. Phase-aligned and phase-misaligned controls of equal total weight yield distinguishable outcomes under entropy stress, isolating alignment as a first-class predictor independent of phase distribution.

Method. Construct two arms with identical phase weights but differing alignment: one with all weight on covered axes, one with 35% of weight redirected to a zero-coverage axis. Sweep injected entropy from low to high in five graduations. Measure residual entropy, order-restoration rate, and recovery efficiency.

Expected result. Aligned controls maintain residual entropy at roughly half the misaligned arm under high injection, with statistically significant separation on recovery rate.

#### 8.2.3. Hardware-Instrumented Landauer Bound Test

Hypothesis. The empirical energetic cost of a React-phase control mechanism approaches or exceeds the Landauer floor $E_{\mathrm{Landauer}}=kT\ln2$ per erased bit of resolved state uncertainty.

Method. Instrument a real authentication or detection pipeline with hardware energy counters (Intel RAPL, AMD equivalent, or ARM energy probe), measure joules per resolved decision, and compare to the Landauer floor for the information-theoretic content of the decision. Repeat for an Intent-equivalent control such as a prebound capability token or an immutable audit-trail entry.

Expected result. React-phase controls exhibit empirical cost within an order of magnitude of the Landauer floor and scale with the bit content of the measurement. Intent-phase controls are bounded above by amortised setup cost only and do not scale with measurement frequency.

#### 8.2.4. Retrospective Breach Analysis

Hypothesis. Disclosed breaches correlate with prior entropy buildup (configuration drift, patch lag, identity sprawl) and latency gaps (detection delay, response delay), not with the absence of nominal controls.

Method. Apply the entropy-latency decomposition to a curated set of public post-incident reports. The Verizon DBIR catalogue, the ENISA NIS2 incident database, and the CISA Known Exploited Vulnerabilities timeline are all compatible. Score each disclosed breach against the lattice and compute the entropy and latency components observed in the window preceding compromise.

Expected result. Breach incidence correlates with high *S* (drift) and high *L* (detection or response delay) readings, independently of which controls were nominally in place. The correlation is the structural prediction; the absence of correlation, given sufficient sample size, would disconfirm the hypothesis.

### 8.3. Limitations and Future Work

The present validation should be interpreted as simulation-based internal consistency evidence rather than external empirical validation. The architecture comparison tests whether the formal model produces its predicted ordinal relationship under fixed, theory-motivated assumptions. It does not establish a universal physical law, and the reported values of $P_{\mathrm{sim}}$, energy cost, latency, and residual entropy should not be read as direct estimates for deployed systems.

Several limitations follow. A central operational limitation concerns measurement portability. The proposed Persistence Index should not be interpreted as a directly transferable universal score across heterogeneous infrastructures unless the measurement protocol, normalization bounds, and observables are explicitly reported. For each deployment domain, empirical use of *P* requires a measurement profile specifying: (i) the protected and adverse states being compared; (ii) the observables used to estimate Δ(t); (iii) the energy or operational-cost proxies used for $E_{\mathrm{cost}}$; (iv) the latency windows used for L(t); (v) the residual-uncertainty or entropy proxies used for S(t); and (vi) the normalization bounds and sensitivity analysis. Without these definitions, *P* should be interpreted as a within-scenario comparison rather than as an absolute cross-domain metric.

The phase-level energy, latency, and entropy constants are specified by the model rather than measured from hardware instrumentation or production telemetry. The energetic term is a normalized operational cost rather than a joule-level thermodynamic measurement. The simulated threat stress and entropy load simplify adversarial adaptation, operator behavior, supply-chain effects, organizational incentives, and heterogeneous infrastructure. The React overload term models the predicted Zeno-like cost nonlinearity, but alternative functional forms and parameter ranges require sensitivity analysis. The Control Lattice has not yet been validated against independent operational datasets.

The present experiment also does not yet report scalability or robustness curves. In particular, it does not vary the number of assets, number of control points, time horizon, monitoring frequency, attacker complexity, entropy-injection rate, or latency-injection rate. These scalability indicators are necessary before the framework can be generalized from a controlled simulation to large enterprise, IoT, or cyber-physical infrastructures. Future experiments should therefore report how $P_{\mathrm{sim}}$, energy cost, residual entropy, compromise rate, MTTR, and analyst workload change as system size and threat complexity increase.

Future work should therefore proceed along the following empirical paths. The independent groups should replicate the simulation with open code, alternative seeds, and broader parameter sweeps to test the robustness of the ordinal prediction. Hardware-instrumented experiments should measure CPU, memory, and power draw for matched Intent-, React-, and Resolve-phase controls using platform energy counters or external probes. The entropy-injection and latency-injection experiments should stress controlled environments with configuration drift, identity inconsistency, delayed telemetry, and response queueing to test whether *P* degrades as predicted. The retrospective breach studies should map public incident reports to the Control Lattice and estimate whether entropy buildup and latency gaps predict detection delay, recovery burden, or breach severity. The field studies should compare real architectures with similar nominal risk profiles but different temporal control placement to determine whether Intent- and Resolve-heavy designs preserve separation at lower operational cost.

Future work should also include comparative case studies in operationally distinct domains and each case study should compare architectures with similar asset criticality and threat exposure but different temporal control placements. The predicted observation is that architectures with greater Intent and Resolve weight should preserve comparable or greater Δ(t) at lower normalized $E_{\mathrm{cost}}$, L(t), and S(t) than React-heavy designs. Such studies would test whether the simulation-derived ordering persists under real telemetry, human workload, infrastructure heterogeneity, and adversarial pressure.

## 9. Conclusions

Security is not an invention. It is a response. Wherever systems face the risk of disorder or delay, entropy or latency, security emerges as the corrective structure that holds coherence together. Across physics, biology, computation, and society, the act of maintaining separation between what must be preserved and what must be resisted is not optional. It is fundamental.

This paper proposed a quantum-inspired hypothesis that security can be modeled as an information-persistence process: the maintained separation between protected and adverse states under entropy, latency, and control cost. The contribution is not a proof that cybersecurity is governed by a new universal physical law. Rather, it is a formal framing that makes a specific claim testable: architectures that preserve separation through Intent and Resolve should require less costly React measurement than architectures dominated by real-time detection and correction.

The manuscript introduced the Security Persistence Index, the Intent–React–Resolve temporal model, and the 5×3 Control Lattice. Together, these constructs shift the design question from “Which controls are present?” to “Where in time do controls operate, how much separation do they maintain, how much uncertainty remains, and what energetic or operational cost is paid to preserve identity?” The simulation-based architecture comparison provides initial internal-consistency evidence for the proposed Principle of Energetic Asymmetry, but it does not establish external validity.

Accordingly, the provided work should be read as a theoretical and simulation-supported hypothesis rather than as proof of a universal empirical law. Its scientific value lies in making the persistence claim explicit, measurable, and falsifiable; its next test is whether the predicted relationships among separation, energetic cost, latency, and residual entropy hold under hardware-instrumented and field conditions.

## Figures and Tables

**Table 1:** Comparison of the proposed Intent-heavy and React-heavy architectural projections with existing cybersecurity paradigms.

Paradigm or Architecture	Primary Design Question	Dominant Temporal Emphasis	Typical Measurement Basis
Checklist or compliance control inventory	Are required controls present?	Mostly static posture; periodic audit	Control counts, maturity scores, pass/fail evidence
Cyber-risk metrics [10]	Which hazards should be prioritized under uncertainty?	Ex ante assessment and periodic reprioritization	Likelihood, impact, exposure, expected loss
Cyber-resilience [2,3,4,20]	Can the system prepare for, absorb, recover from, and adapt to disruption?	Full life cycle, especially recovery and adaptation	Continuity of function, recovery time, degradation, adaptation capacity
Zero Trust Architecture [1]	Should this subject, device, or action be trusted for this request?	Depends on implementation: Intent if identity and policy are prebound; React if every transaction requires heavy synchronous verification	Access decision quality, policy compliance, authentication/authorization events, session risk
Moving target defense [5]	Can controlled change increase attacker uncertainty?	Intent when variation is preplanned; React when reconfiguration is triggered at run time	Attack-surface diversity, attacker uncertainty, compromise probability, performance cost
Alert-driven SOC and detection-response [8,9]	Can adverse events be detected and contained after contact?	React-dominated	Alert volume, false positives, missed events, mean time to detect/respond, analyst load
Intent-heavy architecture in this paper	Can asset–threat separation be prepared, restricted, and later proven with less real-time measurement?	Intent and Resolve, with bounded React	Δ, *E*, *L*, *S*, $P_{\mathrm{sim}}$, CPU load, false positives, latency, residual entropy
React-heavy architecture in this paper	Can separation be maintained mainly through continuous observation, response, and correction?	React-dominated	Same *P*-based variables plus monitoring frequency and response burden

**Table 2:** Phases of Intent–React–Resolve temporal model.

Phase	Function	Energy Cost	System Impact
Intent	Preemptively restricts	Low	Prevents disorder before it manifests
React	Responds in real time	High	Costly and fragile, necessary only under doubt
Resolve	Restores and proves	Moderate	Closes gaps, enables accountability and learning

**Table 3:** Conceptual structure of the Control Lattice.

	Intent Phase (I)	React Phase (R)	Resolve Phase (V)
Characteristics	Identification	Authentication	Non-repudiation
Context	Restriction	Alarm	Remediation
Force	Authorization	Subjugation	Indemnification
Resources	Availability	Containment	Stabilization
Narrative	Confidentiality	Resilience	Integrity

**Table 4:** Aggregate results of the architecture comparison. Values are arithmetic means over N=1000 simulated trials per architecture using seed 20260511. Lower values are better for all metrics except $P_{\mathrm{sim}}$. The displayed operational metrics are output-scale indicators; $P_{\mathrm{sim}}$ is computed from normalized internal $\hat{\Delta }$, $\hat{E}$, $\hat{L}$, and $\hat{S}$ variables.

Architecture	CPU (%)	False Positives	MTTR (min)	Compromise (%)	Residual Entropy	Latency (ms)	Energy Cost	$P_{\mathrm{sim}}$
Intent-heavy	23.38	5.50	54.29	10.79	0.29	46.00	48.70	5.93
Balanced	32.31	10.15	50.05	13.91	0.37	55.66	67.27	4.66
Misaligned	21.68	9.93	60.81	27.80	0.60	60.80	45.35	4.44
React-heavy	48.96	19.09	54.43	16.70	0.44	72.29	102.19	3.45

## Data Availability

The original contributions presented in this study are included in the article and further inquiries can be directed to the corresponding author.

## Appendix A. OSSTMM 4—The Five-Point Process (5PP)

The Five-Point Process (5PP) in OSSTMM is a universal analytical model for decomposing any interaction. It defines five orthogonal categories that describe how systems are observed, influenced, and maintained:

1. Characteristics—The inherent traits of an entity (identity, attributes, behaviors).
2. Context—The environmental and situational conditions governing interaction.
3. Force—The physical and persuasive capabilities, permissions, or prohibitions that affect influence and control.
4. Resources—The materials necessary for function accessible during interaction.
5. Narrative—The information history and state transition storyline associated with the entity.

In this paper, the 5PP is used to construct the 5×3 Control Lattice, where each category intersects with a time phase (Intent, React, Resolve) to create 15 control eigenstates that collectively describe the full field of resistance to entropy and latency.
